# Safety of lumbar puncture in comatose children with clinical features of cerebral malaria

**DOI:** 10.1212/WNL.0000000000003372

**Published:** 2016-11-29

**Authors:** Christopher A. Moxon, Lei Zhao, Chenxi Li, Karl B. Seydel, Ian J. MacCormick, Peter J. Diggle, Macpherson Mallewa, Tom Solomon, Nicholas A. Beare, Simon J. Glover, Simon P. Harding, Susan Lewallen, Sam Kampondeni, Michael J. Potchen, Terrie E. Taylor, Douglas G. Postels

**Affiliations:** From the Institute of Infection and Global Health (C.A.M., T.S.) and Department of Eye and Vision Science, Institute of Ageing and Chronic Disease (I.J.M., N.A.B., S.P.G., S.P.H.), University of Liverpool (S.P.H.), UK; Departments of Epidemiology and Biostatistics (L.Z., C.L.) and Osteopathic Medical Specialties (K.B.S.) and International Neurology and Psychiatry Epidemiology Program (D.G.P.), Michigan State University, East Lansing; Lancaster University (P.J.D.), UK; Department of Paediatrics and Child Health (M.M.) and the Blantyre Malaria Project (T.E.T.), University of Malawi College of Medicine, Blantyre; St. Paul's Eye Unit (N.A.B.), Royal Liverpool University Hospital; School of Medicine (S.J.G.), University of St. Andrews, UK; Kilimanjaro Centre for Community Ophthalmology (KCCO) (S.L.), University of Cape Town, Department of Ophthalmology, OMB Groote Schuur Hospital Observatory, South Africa; Department of Radiology (S.K.), Queen Elizabeth Central Hospital, Blantyre, Malawi; and Department of Imaging Services (M.J.P.), University of Rochester, NY.

## Abstract

**Objective:**

We assessed the independent association of lumbar puncture (LP) and death in Malawian children admitted to the hospital with the clinical features of cerebral malaria (CM).

**Methods:**

This was a retrospective cohort study in Malawian children with clinical features of CM. Allocation to LP was nonrandom and was associated with severity of illness. Propensity score–based analyses were used to adjust for this bias and assess the independent association between LP and mortality.

**Results:**

Data were available for 1,075 children: 866 (80.6%) underwent LP and 209 (19.4%) did not. Unadjusted mortality rates were lower in children who underwent LP (15.3% vs 26.7% in the no-LP group) but differences in covariates between the 2 groups suggested bias in LP allocation. After propensity score matching, all covariates were balanced. Propensity score–based analyses showed no change in mortality rate associated with LP: by inverse probability weighting, the average risk reduction was 2.0% at 12 hours (95% confidence interval −1.5% to 5.5%, *p* = 0.27) and 1.7% during hospital admission (95% confidence interval −4.5% to 7.9%, *p* = 0.60). Undergoing LP did not change the risk of mortality in subanalyses of children with severe brain swelling on MRI or in those with papilledema.

**Conclusion:**

In comatose children with suspected CM who were clinically stable, we found no evidence that LP increases mortality, even in children with objective signs of raised intracranial pressure.

Cerebral malaria (CM)—defined as coma, *Plasmodium falciparum* parasitemia, and the exclusion of other causes of encephalopathy—is a leading cause of death in sub-Saharan Africa.^1,2^ CNS coinfections are well recognized in these patients,^3^ and can only be reliably excluded by lumbar puncture (LP).

Despite its diagnostic utility, performing LPs in children with suspected CM is controversial because the disease may cause raised intracranial pressure (ICP).^2^ Brain swelling has recently been shown to be an independent predictor of death in CM.^4^ Some physicians are concerned that LP in the context of diffusely increased ICP may precipitate fatal herniation^2,5,6^; others believe the procedure is safe in the absence of overt brain shift.^7,8^

Executing a randomized clinical trial to determine if LP is associated with death in children with CM would be problematic. Instead, we carried out a retrospective analysis of children with clinical features of CM in whom LPs were performed unless there were asymmetrical neurologic findings or the admitting clinician believed the child was medically unstable.

To adjust for the selection bias against children who were more severely ill and thus had a higher chance of a fatal outcome, we used propensity score–based analyses.^9^ The propensity score represents the probability, based on observed clinical characteristics, that a participant received the intervention—in this case, an LP. Matching children by propensity score controls for illness severity, in effect simulating a clinical trial. We used this approach to assess the association between LP and death within 12 hours of admission and during the entire hospitalization.

## METHODS

We studied comatose (Blantyre Coma Score ≤2) children aged 6 months to 16 years, admitted to a study investigating CM pathogenesis on the Pediatric Research Ward, Queen Elizabeth Central Hospital, Blantyre, Malawi, from January 1997 to June 2013.

Children with neck stiffness had an LP performed prior to research ward admission. Those with visibly cloudy CSF were enrolled in studies on meningitis treatment, were not enrolled in the parent study, and are not included here. Children discovered to have meningitis after research ward admission were included.

LPs were performed using 21-gauge needles in children deemed medically stable by the admitting clinician, following informed parental consent. LPs were performed either before research ward admission (in the emergency department or general pediatric ward) or on the research ward within 30 minutes of admission. Twelve-hour mortality was determined from the time of LP (in those who underwent LP) or from the time of research ward admission (in those who did not undergo LP). Children had a funduscopic examination by the admitting clinician and if papilledema was suspected, an LP was not performed. Following admission investigations, including LP, definitive funduscopic examination was performed by an ophthalmologist; the ophthalmologist's determination of the presence or absence of papilledema was used in our analyses. LPs were done in children with papilledema when it was recognized by the ophthalmologist after LP performance.^10^ The ophthalmologist's funduscopic examination also assessed for characteristic retinal changes that are a sensitive and specific indicator of the presence of sequestered malaria-infected erythrocytes in the brain.^11^ This assessment distinguishes children with retinopathy-positive CM from those with retinopathy-negative CM, who are more likely to have a nonmalarial cause of coma.^11^

Parasitemic patients were treated with IV quinine. Adjunctive ceftriaxone was given until clinical recovery to all children in whom an LP was omitted. Among children who received an LP, aparasitemic children and children with retinopathy-negative CM were given empirical ceftriaxone pending blood cultures, whereas children with retinopathy-positive CM were only given ceftriaxone if there was significant CSF pleocytosis (leukocytes >30/μL), signs of sepsis, or fever following parasite clearance.

Beginning in 2009, after LP, children who were clinically stable underwent brain MRI with a 0.35T Signa Ovation Excite scanner (GE Healthcare, Milwaukee, WI) within 12 hours of admission. MRI scans were independently interpreted by 2 radiologists.^12^

### Study protocol approvals, registrations, and patient consents.

The parent study's protocol was approved by the University of Malawi College of Medicine Research Ethics Committee and the Biomedical Institutional Review Board of Michigan State University.

### Statistical analysis.

Propensity score calculations included characteristics likely to influence a clinician's decision to perform an LP and those previously established as being associated with fatal outcome, including the following:

1. Depth of coma (Blantyre Coma Score)
2. Systolic blood pressure for age
3. Weight-for-height *z* score (nutritional status)
4. Respiratory distress or acidotic breathing
5. Pulse rate
6. Cardiovascular system examination (signs of heart failure)
7. Sex
8. Admission blood glucose concentration
9. Peripheral parasite density
10. Hematocrit
11. Malarial retinopathy status
12. Papilledema

The various propensity score–based analysis methods each have strengths and weaknesses. To ensure methodologic rigor, we used 3 methods to assess the independent association of LP with mortality: (1) inverse probability weighting, (2) unconditional logistic regression where propensity score was an independent variable, and (3) nearest neighbor propensity score matching (with replacement).^13^ We imputed retinopathy status in 456 participants in whom this variable was missing using logistic regression based on predictive characteristics: platelet count, hematocrit, and glucose.^14^ In participants where the calculated likelihood of being retinopathy-positive was greater than 50%, participants were imputed to be retinopathy-positive; if not, they were imputed as being retinopathy-negative. Papilledema was considered an important covariate for the calculation of propensity score but its determination by an ophthalmologist (considered the gold standard) was missing in a significant number of children. We therefore performed analyses both with papilledema (to account for the variable) and without it (to maximize study power) included in the calculation of propensity score. Other missing data were handled by listwise deletion. In all analyses, a *p* value <0.05 was considered statistically significant. A detailed description of the statistical methods used in our analyses may be found in appendix e-1 at Neurology.org.

## RESULTS

### Characteristics of the patients and balance before and after propensity score matching.

**Figure 1:**
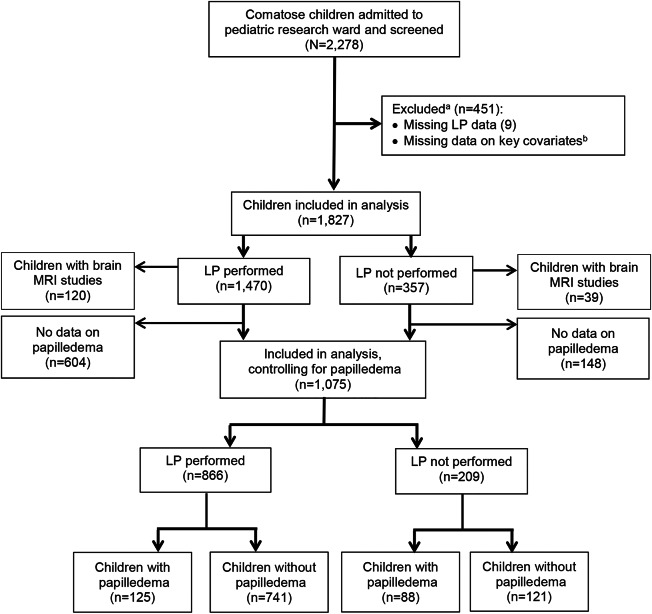
Study populations for propensity score–based analyses ^a^ Compared to those included, the 451 children excluded from analysis did not differ by sex, age, or mortality rate (using a threshold of a 10% difference as clinically meaningful). ^b^ Covariates missing (number of children and covariate): 37 malarial retinopathy, 214 respiratory distress, 67 peripheral parasite density, 27 hematocrit, 90 blood pressure, 27 heart examination, 11 pulse, 10 glucose, 4 sex, 5 age (252 children have missing malarial retinopathy in original data, among which retinopathy status of 215 children are imputed from a logistic regression with hematocrit, platelet count, glucose as covariates: retinopathy status imputed as positive if predicted probability >0.5). LP = lumbar puncture.

**Figure 2:**
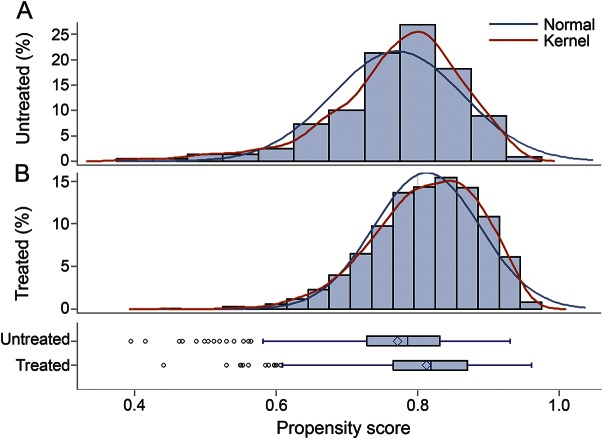
Distribution of propensity scores in those who did not (A) and did (B) receive lumbar punctures Overlap of curves demonstrates that propensity score matching analyses are feasible.

A total of 2,278 comatose patients were admitted to the research ward between January 1997 and June 2013. Of these, 451 children were excluded: 442 had missing data on one or more covariates used in the calculation of propensity score (after retinopathy status imputation); 9 had missing data on LP performance (figure 1). Mortality rates in participants included in our analyses were not different, compared to those excluded due to missing covariates (*p* = 0.34 for 12-hour mortality and 0.50 for mortality during hospitalization). In the analysis omitting papilledema from the calculation of propensity score, 1,827 patients were included: 1,470 (80.5%) of these patients received an LP and 357 (19.5%) did not. In the analysis that included papilledema, 1,075 patients were included, of whom 866 (80.6%) had an LP and 209 (19.4%) did not. Propensity scores of children who did and did not undergo LP overlapped significantly, indicating that propensity score matching analyses were feasible (figure 2). Forty-one of the 866 children who underwent LP (4.7%) had CSF pleocytosis (leukocytes >10); a bacterial pathogen was isolated from CSF in 20 cases and tuberculous meningitis was diagnosed in 3 patients. A total of 87.6% (942 of 1,075 children) had malaria parasitemia; of these, 67.9% were malarial retinopathy–positive.

Before propensity score matching, a number of baseline characteristics were not balanced between children who did and did not undergo LP, as indicated by standardized differences greater than 0.1 (10%) in these variables^15^ (tables 1 and 2). In the analysis in which papilledema was included in the calculation of propensity score, the 1,075 matched pairs generated had acceptable balance for all baseline characteristics (table 1). When papilledema was excluded from the calculation of propensity score, the 1,827 matched pairs were balanced for all baseline characteristics except papilledema, which in those children with data on this covariate was more than twice as frequent in children who did not receive an LP than in those who did (38% vs 16%; standardized difference 53%; table 2).

**Table 1:**
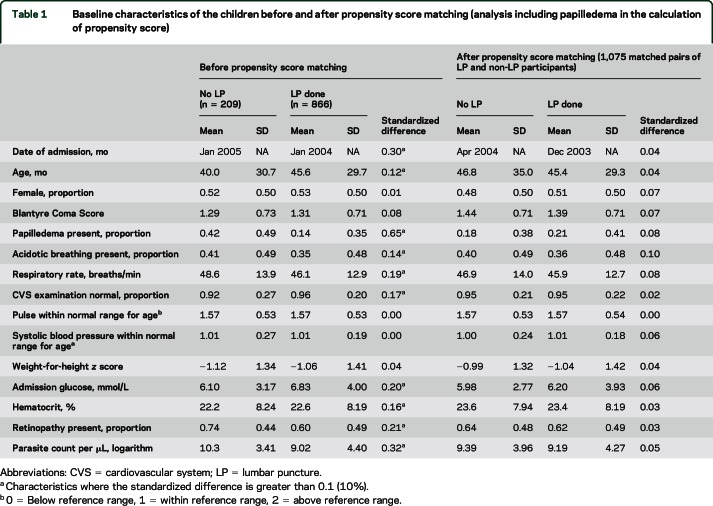
Baseline characteristics of the children before and after propensity score matching (analysis including papilledema in the calculation of propensity score)

**Table 2:**
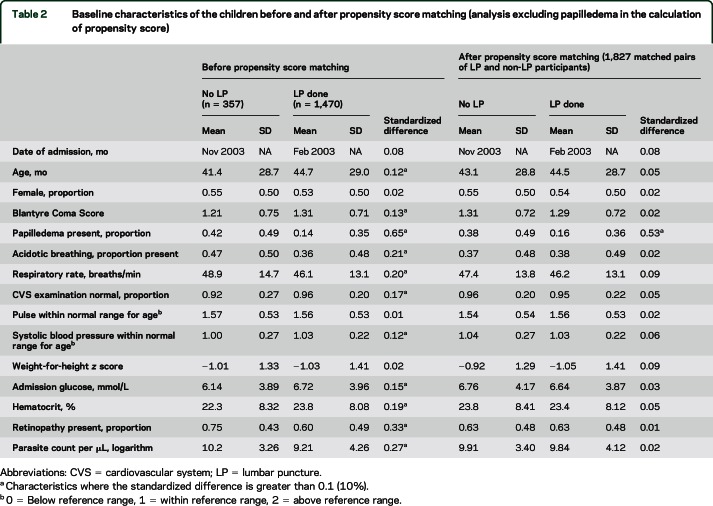
Baseline characteristics of the children before and after propensity score matching (analysis excluding papilledema in the calculation of propensity score)

Since the presence of papilledema influenced whether or not a child received an LP, we report our findings from the analyses where papilledema was included in propensity score calculation. Results of the analyses where papilledema was not used in propensity score calculation are presented in table e-1. We performed separate analyses for patients suspected to have CM on presentation (Table 3), as well as those who strictly fulfilled WHO clinical criteria (coma malaria parasitemia, no other coma etiology evident [table e-2]), and both showed similar results.

The proportion of children who were HIV-positive was balanced in the LP vs no LP groups after propensity score matching: 12.2% vs 9.6% (standardized difference 0.08) in the analysis including papilledema in propensity score calculation and 13.1% vs 12.5% (standardized difference 0.02) in the analysis excluding papilledema.

### Association of LP with mortality at 12 hours.

Unadjusted 12-hour mortality was 7.9% lower in children who received an LP compared to those who did not (95% confidence interval [CI] 5.2%–10.7%, *p* < 0.0001; table 3). In the propensity score–adjusted analysis, 12-hour mortality was not significantly different in children who underwent LP compared to those who did not (table 3). This was the case regardless of the propensity score–based analysis method used. The effect size was similar and insignificant in all 3 statistical methods.

**Table 3:**
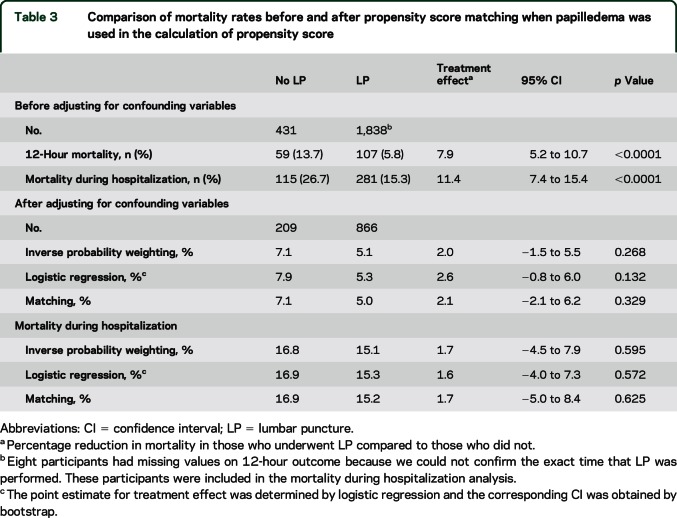
Comparison of mortality rates before and after propensity score matching when papilledema was used in the calculation of propensity score

### Association of LP with mortality during hospitalization.

Unadjusted overall mortality was 11.4% lower in the children who received LPs compared to those who did not (95% CI 7.4%–15.4%, *p* < 0.0001; table 3). After adjustment for propensity score, children who did and did not receive LPs had similar mortality rates (table 3). The effect size was similar in all 3 statistical methods. For children with WHO clinically defined CM, there was no significant difference in adjusted overall mortality rates for those who underwent LP, compared to those who did not (table e-2).

### Influence of papilledema on mortality after LP.

To determine whether the presence of papilledema changed the association of LP with mortality, we assessed interactions between papilledema and LP in our inverse probability–weighted regression analyses. In the model where outcome was mortality at 12 hours, the effect of LP on mortality was not different when comparing children with or without papilledema (difference in treatment effect 5.9%, 95% CI −1.4% to 13.2%, *p* = 0.12). Similar results were obtained for overall mortality (difference in treatment effect 8.8%, 95% CI −2.5% to 20.2%, *p* = 0.13), indicating no effect modification by papilledema.

### Association of LP with mortality in children with severely increased brain volume on MRI.

A total of 166 children underwent brain MRI scans after LP performance. Three children died within 12 hours of admission: 1 had an LP and the other 2 did not. Overall mortality of children who underwent MRI was 12.7% (n = 21).

When restricting our subanalysis to the 101 children who had severe brain swelling with loss of brain sulci and cisterns, with or without evidence of herniation, mortality during hospitalization was 17.1% lower in the children who received an LP compared to those who did not (95% CI −1.4% to 35.6%, *p* = 0.07, inverse probability weighting). Mortality within 12 hours was lower in children who underwent LP by 6.3% (95% CI −1.9% to 14.5%, *p* = 0.13). To maximize the power of this subanalysis, we did not use papilledema in the calculation of propensity score.

## DISCUSSION

In this large single-center study of comatose African children without overt meningismus but with suspected CM, we found no evidence that those undergoing LP were at increased risk of death. This was true even in a subanalysis of children with MRI evidence of severe brain swelling, several of whom had early evidence of herniation. While both papilledema and brain swelling on MRI are independently associated with fatal outcome in CM,^4,16^ in children lacking lateralizing signs and judged to be stable enough to tolerate LP by the admitting clinician, undergoing LP did not change mortality risk in patients with these abnormalities.

The independent association of LP and death in CM or other CNS infections that diffusely raise ICP has not been studied.^7^ Case series of patients with meningitis^5^ and CM^17^ have described temporal associations between LP and death, but fatal herniation in the absence of LP is recognized in both conditions.^5,7^ Since these case series included few patients with diffuse brain swelling who did not undergo LP, they were unable to assess the independent association between LP and outcome. By contrast, our cohort included large groups of children with diffuse brain swelling who did and did not undergo LP, allowing us to test the independent effect of LP on outcome using propensity-score matching. Mortality rates were high in both those who underwent LP and those who did not. We found no evidence that undergoing LP independently increased the risk of death.

These findings are in keeping with experiments investigating CSF dynamics in human cadavers where rapid equilibration between intraspinal and intracranial compartments was seen when LP was performed.^17^ Failure of equilibration and herniation occurred only when free CSF movement was obstructed. We surmise that LP does not exacerbate herniation in CM because, during LP, the CSF pressure is able to rapidly equilibrate. It is important to note that this may not be the case in patients with raised ICP of different etiologies (e.g., space-occupying lesion), which may impair the free movement of CSF.

CM is the most common cause of encephalopathy in sub-Saharan Africa and is therefore a differential diagnosis in a high proportion of children with suspected brain infection. If clinicians are concerned that LP is not safe in this clinical scenario, LPs are likely to be omitted. Omission of LPs leads to empirical antimalarial and antibiotic administration with suboptimal management through both undertreatment and overtreatment. Frequently, children with suspected CM recover within 24 hours of initiating treatment. If both antibiotics and antimalarials are given and an LP has not been performed, the underlying etiology (meningitis, CM, or both) is difficult to determine. Performing an LP after recovery of consciousness and partial treatment is suboptimal because in malaria-endemic settings, molecular diagnostics are rarely available. If an LP is not done at admission, one clinical option is to provide the full course of antibiotic and antimalarial treatment to all children, resulting in many children receiving unnecessary IV drugs, experiencing longer hospital stays, and placing strain on highly limited resources. Alternatively, clinicians could stop medications and discharge the patient, risking incomplete treatment. Epidemiologically, omitting LPs obscures recognition of changes in prevalence of relevant pathogens. On a population level, the cumulative effects of these suboptimal situations are substantial.

Though retrospective, our study has several strengths. We included data collected over 16 years, achieving a sample size that would have been difficult to accrue in a prospective trial. We included funduscopic examinations performed by ophthalmologists and MRI scans interpreted by neuroradiologists, creating an extensive dataset including children with objective signs of increased ICP.

This study has several limitations. We did not assess the impact of LP on neurologic morbidity in survivors. It is possible that LP caused neurologic impairment but did not lead to death, although it is difficult to think of a mechanism whereby this would systematically occur. Although propensity score matching is a powerful and widely used method for reducing bias in observational studies, it has statistical limitations, as do all analytical techniques.^18^ Because the parent study focused on CM pathogenesis, the majority of patients included in our study had CM. Children with meningismus and a visibly cloudy CSF were included in a separate study on meningitis; because they lacked comparable follow-up data and key covariates for propensity score matching, they were not included in this analysis. The 4.7% rate of CSF pleocytosis in our cohort is likely an underestimate of the true frequency of concurrent meningitis in children with suspected CM.

Given that increased brain volume is on the causal pathway to death in children with CM,^4^ children with this condition are a useful group in whom to assess the longstanding controversy as to whether LP has any causal role in fatal herniation in the context of diffusely increased ICP. Our data provide evidence that it does not. Although it remains to be determined whether our findings are generalizable to meningitis or other conditions that cause diffusely raised ICP, they challenge 2 of the common rationales for eschewing LPs in comatose children: that the temporal association between LP and death implies causation and that LP contributes to mortality in children with CM who have increased ICP. Mortality rates were high in children shortly after admission, whether or not they underwent LP. Among patients with objective evidence of increased ICP, LP had no independent association with mortality.

In the clinical scenario of children with suspected CM who are medically stable and lack localizing neurologic signs, our findings provide reassurance to clinicians that they may safely use this important diagnostic tool.

## EDITOR'S NOTE

Please see the Editor's Blog about this article on our special interest site, Without Borders, at wb.neurology.org.

## Supplementary Material

Data Supplement

## GLOSSARY

CI: confidence interval
CM: cerebral malaria
ICP: intracranial pressure
LP: lumbar puncture
