# Supplementary material for: Safety of lumbar puncture in comatose children with clinical features of cerebral malaria
Source: Neurology. 2016 Nov 29;87(22):2355–62. doi: 10.1212/WNL.0000000000003372 (PMC5135026; doi:10.1212/WNL.0000000000003372)
Supplement: Data Supplement [file supp_87_22_2355__index.html]

Safety of lumbar puncture in comatose children with clinical features of cerebral malaria — Data Supplement 

# Safety of lumbar puncture in comatose children with clinical features of cerebral malaria

## Data Supplement

**Neurology® data supplements are not copyedited before publication. Published editorials and translations have been copyedited.  
 © 2016 American Academy of Neurology.  
  
 Files in this Data Supplement:**

- Data Supplement - Microsoft Word file
