# Supplementary material for: Safety of lumbar puncture in comatose children with clinical features of cerebral malaria
Source: Neurology. 2016 Nov 29;87(22):2355–62. doi: 10.1212/WNL.0000000000003372 (PMC5135026; doi:10.1212/WNL.0000000000003372)
Supplement: Data Supplement [file supp_WNL.0000000000003372_Supplemental_Files.docx]

**Appendix e1: Statistical Analysis Methods for ‘Safety of Lumbar Puncture in Comatose Children With Clinical Features of Cerebral Malaria’**

Given the complexity of the statistical analysis in this paper and in order to facilitate reproducibility of our study results, we describe the analytic process and methods in detail in this supplement.

Propensity score (PS) methods are a set of statistical techniques that attempt to estimate the causal effect of a treatment by accounting for the covariates that predict both the outcome and whether to receive the treatment. These methods aim to decrease the bias caused by the confounders found in the study especially when there are limited amount of observations but many confounders. In our study, 13 factors including age, gender, Blantyre coma score, papilledema, acidotic breathing, cardiovascular examination, pulse, systolic blood pressure, weight-for-height z score, admission glucose, hematocrit, malarial retinopathy status, and peripheral parasite density were considered confounders, and may have affected both mortality and the decision to perform a lumbar puncture.

**Construction of the analysis sample:**

From the original dataset, we analyzed data from participants whose treatment information was not missing. In order to calculate the propensity score, we required that the confounders (mentioned above) were not missing. In the original dataset, 252 participants were missing an ophthalmologist’s determination of malarial retinopathy status. We imputed retinopathy status with logistic regression using hematocrit, platelet count, and glucose as covariates. Retinopathy status was imputed based on the predicted probability from the logistic regression model. Subjects were imputed to be retinopathy positive if the predicted probability value was greater than 0.5 and were imputed to be retinopathy negative if the predicted probability was less than 0.5.

**Propensity score calculation:**

Papilledema was considered an important covariate for the calculation of propensity score but its determination by an ophthalmologist (considered the gold standard) was missing in a significant number of children. We therefore calculated propensity scores in two scenarios: one with papilledema considered as a confounder (to account for the variable) and one without (to maximize study power). The propensity scores were calculated from a logistic regression model in which treatment is predicted by the confounders. The estimated probability of having a lumbar puncture preformed is the propensity score. Therefore, the propensity score without papilledema being considered (PS1) was estimated from 12 confounders and the propensity score adjusted for papilledema (PS2) was estimated from all 13 confounders. As displayed in Figure 1, 1827 subjects had PS1 calculated and 1075 had PS2 calculated. All the results of the analysis without papilledema being considered as a confounder are based on PS1 and the results of the analysis with papilledema being considered as a confounder are based on PS2.

**Propensity score based methods:**

Statistically, we assumed that the treatment (lumbar puncture) is independent of the counterfactual outcomes (12-hour mortality and overall mortality) after adjusting for the confounders. The following three propensity score based methods were used.

*Inverse probability weighting method:*

The estimated inverse probability weights are the inverse propensity scores if receiving lumbar puncture and the inverse of one minus propensity score if not receiving lumbar puncture. These weights are used to compute the weighted average of outcomes for each treatment. Then the average treatment effects are computed from the difference among these weighted average of outcomes (Lunceford and Davidian, 2004, equation (8) and its variance formula therein).

*Regression adjustment:*

The propensity score was used as a covariate along with a treatment indicator in a logistic regression equation with death as the dependent variable. By doing this, at each treatment level the averages of predicted outcome from each subject can be computed. By taking the difference between the treatment-specific averages we obtained the average treatment effect. We used bootstrapping to compute the confidence interval for this average treatment effect. In this approach, 50 bootstrap samples were obtained by resampling, with replacement, from the observations in the analysis sample. In each bootstrap sample, propensity scores are re-calculated and treatment-specific averages are re-computed. Therefore, we obtained the average treatment effect from each of these samples and used their sample variance as the estimated variance of the average treatment effect estimate based on the analysis sample.

*PS matching:*

The goal of this approach is to form similar groups of treated and untreated subjects with respect to the confounders’ distributions by matching each subject with a subject in the opposite treatment group based on the propensity score. In this study, we use one-to-one matching with replacement. The nearest neighbor method was used to create pairs: e.g. for a subject A in treated group whose propensity score is 0.8, subject B is selected by identifying the subject in the untreated group whose propensity score is closest to 0.8 among all the untreated. We then repeated the process for all other treated subjects. Similarly, we performed matching for untreated subjects. The average treatment effect was computed by taking the average of differences between the pairs. For inference, we used the variance formula for the above matching estimator derived in Abadie and Imbens (2016).

**Subgroup analysis:**

*Influence of papilledema on mortality after lumbar puncture:*

We fit a marginal structural model with lumbar puncture, papilledema and their interaction term as covariates and weights being inverse probabilities of treatment based on the propensity scores. The inference for the interaction term’s coefficient helps to determine if there is effect modification by papilledema to lumbar puncture’s effect on mortality.

*Association of LP with mortality in children with severely increased brain volume on MRI:*

In this subgroup analysis, we only included 101 subjects who had the most severe category of brain swelling (loss of brain sulci and cisterns shown by MRI scans, with or without early herniation). To maximize the power of this subgroup analysis, we did not use papilledema in the calculation of propensity score. The inverse probability weighting method was used to estimate the effect of lumbar puncture on mortality in this sub-population.

**Implementing software:**

All propensity score calculations were performed with SAS. The other statistical analyses were performed using STATA.

**References:**

Lunceford, J. K., & Davidian, M. (2004). Stratification and weighting via the propensity score in estimation of causal treatment effects: a comparative study. *Statistics in medicine*, 23(19), 2937-2960.

Abadie, A., & Imbens, G. W. (2016). Matching on the estimated propensity score. *Econometrica*, 84(2), 781-807.

**Supplemental Table e-1:** Comparison of mortality rates before and after propensity score matching when papilledema is not used in the calculation of propensity score

| **Before adjusting for confounding variables** | | | | | |
| --- | --- | --- | --- | --- | --- |
|  |  |  | Treatment effect^2^ | 95% confidence interval (CI) | P value |
|  | No LP | LP |  |  |  |
| Number | 431 | 1838^1^ |  |  |  |
| ***12 hour mortality*** | 59 (13.7%) | 107 (5.8%) | 7.9 | 5.2, 10.7 | <0·0001 |
| ***Mortality during hospitalization*** | 115 (26.7%) | 281 (15.3%) | 11.4 | 7.4, 15.4 | <0·0001 |
|  | | | | | |
| **After adjusting for confounding variables** | | | | | |
|  | No LP | LP |  | | |
| Number | 357 | 1470 |  | | |
| ***12 hour mortality*** |  |  | Treatment effect^2^ | 95% CI | P value |
| Inverse probability weighting | 10.5% | 5.9% | 4.6 | 1.4, 7.8 | 0·005 |
| Logistic regression^3^ | 10.5% | 5.9% | 4.6 | 1.3, 7.9 | 0·007 |
| Matching | 11.9% | 6.9% | 4·9 | 0.1, 9.8 | 0·046 |
| ***Mortality during hospitalization*** |  |  |  |  |  |
| Inverse probability weighting | 22.5% | 15.4% | 7.1 | 3.2, 11.0 | 0·001 |
| Logistic regression^3^ | 23.0% | 15.4% | 7·6 | 3.0, 12.3 | 0·001 |
| Matching | 22.1% | 15.4% | 6·7 | 0.4, 13.1 | 0·038 |

1. 8 subjects had missing values on 12 hour outcome because we could not confirm the exact time that lumbar puncture was performed. These subjects were included in the mortality during hospitalization analysis.

2. Percentage reduction in mortality in those who underwent LP compared to those who did not

3. The point estimate for treatment effect was determined by logistic regression and the corresponding confidence interval was obtained by bootstrap

**Supplemental Table e-2:** Comparison of mortality rates before and after propensity score matching when papilledema was used in the calculation of propensity score. Analysis restricted to children fulfilling WHO diagnostic criteria for cerebral malaria

| **Before adjusting for confounding variables** | | | | | |
| --- | --- | --- | --- | --- | --- |
|  |  |  | Treatment effect^2^ | 95% confidence interval (CI) | P value |
|  | No LP | LP |  |  |  |
| Number | 406 | 1635^1^ |  |  |  |
| ***12 hour mortality*** | 53 (13.1%) | 90 (5.5%) | 7.6 | 4.8, 10.4 | <0.0001 |
| ***Mortality during hospitalization*** | 105 (25.9%) | 235 (14.4%) | 11.5 | 7.5, 15.5 | <0.0001 |
|  | | | | | |
| **After adjusting for confounding variables** | | | | | |
|  | No LP | LP |  | | |
| Number | 196 | 750 |  | | |
| ***12 hour mortality*** |  |  | Treatment effect^2^ | 95% CI | P value |
| Inverse probability weighting | 7.3% | 4.7% | 2.6 | -1.3, 6.5 | 0.198 |
| Logistic regression^3^ | 7.0% | 4.8% | 2.2 | -1.3, 5.6 | 0.213 |
| Matching | 6.8% | 4.1% | 2.6 | -1.4, 6.7 | 0.199 |
| ***Mortality during hospitalization*** |  |  |  |  |  |
| Inverse probability weighting | 15.4% | 13.9% | 1.5 | -4.2, 7.1 | 0.610 |
| Logistic regression^3^ | 15.4% | 13.9% | 1.5 | -4.0, 7.0 | 0.595 |
| Matching | 13.6% | 14.2% | -0.5 | -6.3, 5.3 | 0.858 |
